# Identification of Peripheral Fatigue through Exercise-Induced Changes in Muscle Contractility

**DOI:** 10.5114/jhk/185297

**Published:** 2024-05-17

**Authors:** Francisco Piqueras-Sanchiz, Saul Martin-Rodriguez, Pedro J. Cornejo-Daza, Juan Sánchez-Valdepeñas, Virginia Serrano-Gómez, Fernando Pareja-Blanco, Óscar García-García

**Affiliations:** 1 Department of Sports and Computers Sciences, Physical Performance & Sports Research Center, Universidad Pablo de Olavide, Sevilla, Spain.; 2 Department of Physical Education, University of Las Palmas de Gran Canaria, Las Palmas de Gran Canaria, Spain.; 3 Department of Sports and Computers Sciences, Faculty of Sport Sciences, Universidad Pablo de Olavide, Sevilla, Spain.; 4 Sport Performance, Physical Condition and Wellness Lab. Faculty of Education and Sport Sciences, Universidad de Vigo, Pontevedra, Spain.

**Keywords:** tensiomyography, resistance training, maximal isometric force, countermovement jump, muscle contractile properties

## Abstract

The aim of this study was to assess whether tensiomyography is a tool sensitive enough to detect peripheral fatigue. Twenty-six strength-trained men were split into two groups: 1) a fatigued group (FG), who performed a full-squat (SQ) standardized warm-up plus 3 x 8 SQs with 75% 1RM with a 5-min rest interval, and 2) a non-fatigued group (NFG), who only did the SQ standardized warm-up. The countermovement jump (CMJ), maximal isometric force (MIF) in the SQ at 90º knee flexion, and TMG in vastus medialis (VM) and vastus lateralis (VL) muscles were assessed pre- and post-protocols. Data were analyzed through mixed ANOVA, logistic regression analysis, and receiver-operating curves. There were significant group x time interactions (p < 0.01) for CMJ height, MIF, maximal radial displacement (Dm), and radial displacement velocity (Vrd_90_) since the FG acutely decreased in these variables, while no significant changes were observed for the NFG. The logistic regression showed a significant model for detecting fatigue, whether it used the CMJ or MIF, with only the relative change in VL-Vrd_90_ as a fatigue predictor. The determination of the area under the curve showed that Dm and Vrd_90_ had good to excellent discriminative ability. Dm and Vrd90 are sensitive to detect fatigue in VL and VM muscles in resistance training contexts.

## Introduction

Peripheral fatigue, acknowledged for its impact on skeletal muscle force generation (Kirkendall, 1990), extends its influence downstream of the neuromuscular junction, affecting electrochemical and mechanical mechanisms crucial to force transmission at the tendon insertion point (Westerblad, 2016). The consequential ramifications of fatigue on athletic performance (Morales-Alamo et al., 2015) and the heightened risk of sports-related injuries (Jones et al., 2017), underscore the significance of its comprehensive study. Recent advancements in technology and methodologies have significantly broadened our understanding of the electrochemical and mechanical factors contributing to force output impairment during and post-fatiguing tasks (Cé et al., 2020). Considering various innovative technologies, such as laser diffraction, 31P magnetic resonance spectroscopy, shear-wave elastography, myotonometry, mechanomyography, and high-density surface electromyography, have been employed to investigate the effects of peripheral fatigue linked to skeletal muscle contraction, their mechanistic insights into the electrochemical and mechanical aspects of fatigue have been reviewed (Cé et al., 2020). Within this array of technologies, tensiomyography (TMG) has emerged as a widely utilized tool, with over 170 articles in PubMed since the 1990s, particularly in assessing alterations in muscle contractile properties for the evaluation of fatigue or recovery processes (Macgregor et al., 2018).

Although TMG technology is widely used in elite sports teams to monitor fatigue, there is hardly any literature on the topic, validating TMG as a tool to indirectly measure peripheral fatigue (Kalc et al., 2023; Martín-San Agustin et al., 2020; Wiewelhove et al., 2017). Except for these articles, most of the literature in this regard consists of observational studies with different interventions that induce fatigue and in which the changes in these variables are evaluated at the same time as they are compared with indirect tests to assess fatigue (Buoite Stella et al., 2023; Cuba-Dorado et al., 2023; García-García et al., 2020; García-Manso et al., 2011; García-Sillero et al., 2021; Gutiérrez-Vargas et al., 2020; Muñoz-López et al., 2022; Pakosz et al., 2023; Piqueras-Sánchiz et al., 2022; Pereira et al., 2020; Raeder et al., 2016). Results from these studies are controversial since the direction of the changes in the TMG variables (i.e., maximal radial displacement (Dm), time contraction (Tc), radial displacement velocity (Vrd_90_)) are intervention dependent, thus there is no clear variable indicating fatigue. In this regard, Wiewelhove et al. (2017) were the first to attempt to resolve whether TMG could indirectly detect fatigue, and for this, they used a high-intensity interval training (HIT) protocol in children. Those authors assessed the diagnostic characteristics of TMG with a receiver-operating curve (ROC) analysis and a contingency table, examining the AUC, Youden's index, sensitivity, specificity, and diagnostic effectiveness. They concluded that TMG was not sensitive to detect significant muscular performance changes and consequently, any muscle fatigue induced by a HIT protocol. However, a letter to the editor (Martín-Rodriguez et al., 2017) was written due to several methodological drawbacks. Bayesian calculations allowed to confirm Type II error and indicated that Wiewelhove et al. (2017) should have performed ad hoc power analysis to be confident about their results. In addition, the measures implemented to assess muscle fatigue (lower limb muscle soreness and countermovement jump (CMJ) height) likely reflected significant fatigue of other muscles, but not one of the measured muscles (i.e., rectus femoris (RF)) (Pereira et al., 2019). CMJ performance is not limited by RF muscle fatigue, since Wong et al. (2016) showed that RF plays a marginal role during the push-off phase of vertical jumping and other muscles (vastus lateralis (VL), gastrocnemius, hamstrings) are more important for maximizing jump height.

On the other hand, Martín-San Agustin et al. (2020) demonstrated that Dm and radial displacement velocity of the RF had an acceptable response to peripheral fatigue, using maximal voluntary contraction (MVIC) to assess muscle fatigue. Also, Kalc et al. (2023) evaluated the diagnostic accuracy of TMG variables for monitoring peripheral neuromuscular fatigue after two tasks: a 25% maximal voluntary contraction (MVIC) and a 30-s all-out cycling test. Those authors found that TMG variables showed good diagnostic efficacy in detecting peripheral fatigue, whereas in contrast, they showed poor ability to detect neuromuscular fatigue.

Despite more than a dozen observational articles having merely found how different interventions induced fatigue and modulated TMG variables, few have statistically evaluated the sensitivity of TMG to detect peripheral fatigue (Kalc et al., 2023; Martín-San Agustin et al., 2020; Wiewelhove et al., 2017). In fact, it is still unknown which specific variables can be used as markers of peripheral fatigue based on sensitivity data.

To solve all these questions, the objectives of the present study were: 1) to assess whether TMG is a tool sensitive enough to detect peripheral fatigue compared to reference methods; 2) to determine which TMG variable or variables are more sensitive to changes in peripheral fatigue. We hypothesized that, firstly, TMG would be sensitive to detect fatigue compared to reference methods; and secondly, Dm would be the most sensitive TMG variable to detect peripheral fatigue.

## Methods

### Participants

Twenty-six strength-trained men participated in the study. They were split into two groups: 1) a fatigued group (FG) (n: 16; age: 23.8 ± 4.4 years; body height: 1.75 ± 0.05 m; body mass (BM): 74.6 ± 8.9 kg; one-repetition maximum (1RM) strength for the SQ exercise normalized per BM: 1.56 ± 0.28)); and 2) a non-fatigued group (NFG) (n: 10; age: 24.9 ± 4.7 years; body height: 1.75 ± 0.05 m; BM: 71.5 ± 5.1 kg; 1RM strength for the SQ normalized per BM: 1.48 ± 0.15)). All participants had at least two years of RT experience in the SQ exercise and were familiarized with the tests carried out. Participants were injury-free. They were fully informed about the procedures, potential risks, and benefits of the study, and they all signed a written informed consent form before the tests. This study was approved by the Ethics Committee of the Universidad de Vigo (protocol code: 03-819; approval date: 20 June 2019) and was conducted following the ethical standards set by the Declaration of Helsinki.

### Measures

#### TMG Measures

TMG was used to assess the contractile properties of the vastus lateralis (VL) and vastus medialis (VM) muscles of the right leg using a specific measuring device (TMG-BMC Ltd, Ljubljana, Slovenia), following a previously described standardized protocol (García-García et al., 2019; Simunic et al., 2011). TMG measures in this trial were: Dm, Tc, and Vrd_90_. Dm was defined as the peak amplitude in the displacement-time curve of the tensiomyographic twitch response; Tc was obtained by determining the time interval from 10% to 90% of Dm; Vrd_90_ was defined as mean velocities of muscle contraction (mm•s^−1^) from the onset of electrical stimulation until 90% of Dm (De Paula et al., 2015). Variables were analyzed for isolated muscles (VL and VM) and as the sum of vastus lateralis and vastus medialis values. All measurements were carried out by the same experienced evaluator and only the curve with the highest Dm value was considered for further analysis (García-García et al., 2020). The absolute and relative reliability of TMG have been reported elsewhere (Martín-Rodriguez et al., 2017)

#### CMJ Measures

An infrared timing system (OptojumpNext, Microgate, Bolzano, Italy) was used for determining jump height. The CMJ was used following a previously described standardized protocol (Piqueras-Sanchiz et al., 2022). The CMJ measures in this trial were the mean height between two trials separated by 10 s.

#### MIF by MVIC Measures

Maximal isometric force (MIF) was defined as the maximal strength value attained during the MVIC in the SQ exercise with participants standing with their knees flexed at 90° (180° = full extension). External forces were collected at a sampling rate of 1,000 Hz with an 80 x 80 cm force plate (FP-500; Ergotech, Murcia, Spain) and processed with specific software (T-Force System; Ergotech, Murcia, Spain). The MVIC was used following a previously described standardized protocol (Piqueras-Sanchiz et al., 2022). The average MIF value in the two attempts was recorded for further analysis.

### Design and Procedures

#### Design

A randomized research design was undertaken to assess the sensitivity of TMG measures to identify alterations in performance based on individual contractile property responses to two different groups: 1) the FG, who performed a full-squat (SQ) standardized warm-up plus 3 x 8 in the full-squat with 75% 1RM with a 5-min rest interval, and 2) the NFG, who only did the SQ warm-up. To compare fatigue development, the CMJ, maximal voluntary isometric contraction in the SQ at 90º knee flexion, and TMG tests were conducted before and after each protocol. All measurements were completed by each participant on the same day. Participants were asked to abstain from any strenuous physical activity for at least two days before attending the study. All sessions took place at a neuromuscular research laboratory under the direct supervision of the researchers and under stable environmental conditions (20ºC and 60% humidity, approximately).

#### Procedures

Participants were requested to lie down for 10 min before starting the TMG measurements and baseline data acquisition. Then, a standardized warm-up was performed by the FG and the NFG before the CMJ and MVIC tests, consisting of 5 min of jogging at a self-selected easy pace, two sets of 10 squats without an external load, five submaximal CMJs, three maximal CMJs, and CMJ testing. Afterward, participants performed two repetitions of MVIC at 70% and 90% of the perceived effort with 30 s of rest in between, which were followed by a MVIC test.

Afterwards, a standardized SQ warm-up was performed by both groups, which consisted of 6-6-4-3 SQ repetitions with 20 kg, 40%, 50%, and 60% 1RM, respectively, with 3-min rest intervals between loads. Relative loads were determined from the individual load-velocity relationship (R^2^ = 0.996 ± 0.002) obtained from a progressive loading SQ test conducted in the previous week following the protocol described by Piqueras-Sanchiz et al. (2022). The SQ was performed on a Smith machine (Multipower Fitness Line, Peroga, Murcia, Spain) with participants starting from an upright position with the knees and hips fully extended, parallel feet and a stance approximately shoulder-width apart, and the barbell resting across the back at the level of the acromion (Piqueras-Sanchiz et al., 2022). Each participant descended in a continuous motion as low as possible (~35–40° knee flexion), then immediately reversed motion and raised back as fast as possible to the upright position.

Next, only the FG performed 3 x 8 SQs with 75% 1RM and a 5-min rest interval between sets. After the NFG completed the warm-up and the fatigued group conducted the warm-up and the resistance exercise protocol, the battery of tests was repeated as follows: CMJ (20 s-Post), MVIC (50 s-Post), and TMG (5 min-Post). This order was chosen to minimize the interference between tests and record valid data, i.e., the acute response to mechanical performance (but not high-fatiguing tests), and TMG after 4–5 min rest.

### Statistical Analysis

A normality test (Kolmogorov-Smirnov test with Lilliefors correction) and a Levene’s test determined that the sample was normal, linear, and homoscedastic. Test-retest absolute reliability for tensiomyography and the countermovement jump were measured by the standard error of measurement (SEM), which was expressed in relative terms through the coefficient of variation (CV). The SEM was calculated as the root mean square of the total mean square intra-subject. Relative reliability was assessed by the intraclass correlation coefficient (ICC) calculated with the one-way random effects model. A two-way factorial analysis of variance (ANOVA) was used to detect changes after protocols. Two factors were included in the mixed ANOVA model: time (changes detected between pre- and post-protocol assessments) as the within-subjects variable, and groups (FG, NFG) as the inter-subject variable. Effect sizes in mixed ANOVA were reported as partial eta square (η^2^_p_) and interpreted as small (0.01), moderate (0.06), or large (0.14). Relative changes (∆) in each variable were calculated as follows: (Post-Pre)/Pre•100. A logistic regression analysis was used to explore the influence of TMG variables (independent variables) on fatigue (dependent variable). A forward stepwise regression model (Forward: LR) was carried out using a maximum likelihood estimation including the TMG variables after a previous data validation and an analysis of residuals. Moreover, ROC curves were used to investigate the diagnostic accuracy of the TMG measures for the assessment of muscle fatigue in comparison to the criterion measure (i.e., CMJ height or MIF). An area under the curve plots the true positive rate (i.e., sensitivity) against the true negative rate (i.e., specificity) to produce an AUC. An area under the curve serves to estimate how high the discriminative power of a test is. The area can have any value between 0.00 and 1.00. Hosmer and Lemeshow (2000) indicated as representative values of the AUC ≤ 0.50 non-discriminating test, 0.50–0.70 lower; 0.70−0.80 acceptable; 0.80−0.90 good; and ≥0.90 excellent. An alpha level of *p* < 0.01 was considered statistically significant. All data were analyzed using SPSS v.24.0 for Windows (SPSS Inc., Chicago, IL, USA).

## Results

Test-retest reliability for TMG measures by the ICC and CV values were as follows: Dm (ICC: 0.99, CV: 5.6%), Tc (ICC: 0.98, CV: 3.4%), and Vrd_90_ (ICC: 0.99, CV: 4.7%). CMJ test-retest reliability values were ICC: 0.99 and CV: 1.9%.

Table 1 shows changes in all variables analyzed (i.e., CMJ, MIF, and TMG-derived variables) following the two protocols carried out (i.e., FG vs. NFG). Significant group x time interactions were observed for all variables analyzed except for Tc in the vastus medialis. The fatigued group showed lower CMJ height than the non-fatigued group at Post. Likewise, the fatigued group showed a significantly impaired MIF at Post, and the non-fatigued group did not, but no significant differences between groups were observed for this variable. Moreover, Tc did not show significant changes for any muscle (i.e., VL, VM, and VL+VM) and condition (i.e., FG and NFG). The fatigued group attained significant reductions in Dm and Vrd_90_ at Post for all muscles (i.e., VL, VM, and VL+VM), while the non-fatigued group did not obtain significant changes for these variables. Furthermore, the FG obtained significantly lower values of Dm and Vrd_90_ for VL and VL+VM at Post compared with the NFG.

**Table 1:** Descriptive values of vertical jump height, maximal isometric force, and muscle contractile properties after performing both protocols.

Variables	FG Pre	FG Post	∆ (%)	NFG Pre	NFG Post	∆ (%)	Group x time interaction (*p*-value)	η^2^_p_
CMJ	37.4 ± 4.7	26.9 ± 3.9^*#^	−27.8	39.4 ± 6.5	37.3 ± 6.0	−5.8	<0.001	*0.584*
MIF	1279.8 ± 233.6	1036.3 ± 267.6^*^	−19.3	1130.2 ± 191.8	1093.9 ± 196.4	−3.1	0.006	*0.258*
VL Tc	21.8 ± 3.7	22.6 ± 3.5	3.3	25.8 ± 2.1	24.2 ± 2.8	−6.4	0.03	*0.182*
VM Tc	21.90 ± 2.83	22.87 ± 3.47	3.7	22.89 ± 2.06	22.67 ± 2.35	−1.1	0.15	*0.088*
VL+VM Tc	43.71 ± 5.55	45.52 ± 5.54	3.6	48.78 ± 3.55	46.91 ± 4.50	−3.9	0.03	*0.157*
VL Dm	6.13 ± 1.62	3.77 ± 1.57^*#^	−37.1	6.23 ± 1.42	5.75 ± 1.32	−7.2	<0.001	*0.279*
VM Dm	8.58 ± 1.63	6.61 ± 1.54^*#^	−22.5	6.95 ± 1.76	7.20± 1.71	3.5	<0.001	*0.384*
VL+VM Dm	14.71 ± 2.55	10.38 ± 2.58^*#^	−29.4	13.18 ± 2.29	12.95 ± 1.89	−0.9	<0.001	*0.415*
VL Vrd_90_	124.3 ± 33.8	75.7 ± 26.1^*#^	−39.1	110.4 ± 20.8	110.6 ± 22.2	−0.2	<0.001	*0.419*
VM Vrd_90_	176.7 ± 34.0	133.9 ± 35.0^*#^	−23.9	140.1 ± 34.1	151.1 ± 35.5	7.1	<0.001	*0.388*
VL+VM Vrd_90_	300.9 ± 59.7	209.5 ± 41.2^*#^	−30.1	250.5 ± 41.0	261.6 ± 41.2	4.3	<0.001	*0.468*

* significant change *p* < 0.01 in the interaction time x group. FG: fatigued group; NFG: non-fatigued group; Pre: baseline values; Post: post-exercise values; VL: vastus lateralis; VM: vastus medialis; VL+VM: sum of VL and VM values; CMJ: countermovement jump (cm); MIF: maximal isometric force (N); Tc: contraction time (ms); Dm: maximum radial displacement of the muscle belly (mm), Vrd_90_: radial displacement velocity (mm•s^−1^); **∆**: relative difference from pre to post (%).^#^ indicates significant differences (*p* < 0.01) between groups at the corresponding time-point. ^*^ indicates significant differences (*p* < 0.01) within-group with respect to Pre-values.

The logistic regression showed a statistically significant model (*p* = 0.001) for detecting fatigue, using countermovement jump performance as a fatigue pattern (Table 2). The adjusted model obtained an adequate quality shown by the Pseudo-R_2_ (R^2^_CS_ = 0.582; R^2^_N_ = 0.798) with only the ∆ VL Vrd_90_ variable. The predictive model correctly classified 92% of cases though, presenting very good sensitivity (93.8%) and good specificity (88.9%). The logistic regression also showed a statistically significant model (*p* = 0.001) for detecting fatigue, using maximal isometric force as a fatigue pattern (Table 3). The adjusted model obtained an adequate quality shown by the Pseudo-R_2_ (R^2^_CS_ = 0.461; R^2^_N_ = 0.624) with only the ∆ VL Vrd_90_ variable. The predictive model correctly classified 84% of cases, presenting good sensitivity (86.7%) and good specificity (80%).

**Table 2:** Logistic regression analysis for predicting fatigue after a resistance training protocol, using countermovement jump (CMJ) height as a fatigue pattern.

Model	B	Std Error	Wald	gl	*p*-value	Exp (B)	95% CI for Exp (B)	
							Inf	Sup
**∆** VL Vrd_90_	−0.179	0.077	5.430	1	0.020	0.836	0.720	0.972
Constant	−2.224	1.237	3.234	1	0.072	0.108		
**Model Summary** *G*^2^ = 21.802; *p = 0.001; X^2^_HL_= 0.475 p= 0.998; R*^2^_cs_ = 0.582; *R^2^_N_* = 0.798								

a. Dependent variable: Fatigue assessed by CMJ height.

b. Predictors: (Constant); **∆** VL Vrd90: relative change from pre- to post in vastus lateralis (VL) radial displacement velocity (Vrd_90_)

**Table 3:** Logistic regression analysis for predicting fatigue after a resistance training protocol, using maximal isometric force (MIF) as a fatigue pattern

Model	B	Std Error		Wald	gl	*p*-value	Exp (B)	95% CI for Exp (B)	
								Inf	Sup
**∆** VL Vrd_90_	−0.109		0.044	6.157	1	0.013	0.896	0.822	0.977
Constant	−3.454		1.473	5.498	1	0.019	0.032		
**Model Summary** *G*^2^ = 15.472; *p =0.001; X^2^_HL_=1.226 p= 0.976; R*^2^_cs_ = 0.461; *R^2^_N_* = 0 .624									

a. Dependent variable: Fatigue assessed by MIF

b. Predictors: (Constant); **∆** VL Vrd90: relative change from pre- to post in vastus lateralis (VL) radial displacement velocity (Vrd_90_)

The determination of the AUC ± 95% confidence intervals (CI) for the VL muscle with CMJ fatigue (Dm: 0.88, 0.74–1.00; Tc: 0.31, 0.09–0.52; Vrd_90_: 0.98, 0.93−1.00) showed that the Dm variable had a good discriminative ability and Vrd_90_ had an excellent discriminative ability. The determination of the AUC ± 95% CI for the VM with CMJ fatigue (Dm: 0.94, 0.84–1; Tc: 0.46, 0.23–0.68; Vrd_90_: 0.98, 0.93–1.00) showed that Dm and Vrd_90_ variables had an excellent discriminative ability. The addition of Dm and Vrd_90_ (VL+VM) exhibited an excellent discriminative ability (0.98, 0.95−1.00; 0.99, 0.97−1.00, respectively). The determination of the AUC ± 95% CI for the VL with MIF fatigue (Dm: 0.85, 0.70–1.00; Tc: 0.31, 0.07–0.53; Vrd_90_: 0.91, 0.80−1.00) showed a good and an excellent discriminative ability for Dm and Vrd_90,_ respectively. The determination of the AUC ± 95% CI for the VM with MIF fatigue (Dm: 0.86, 0.72–1; Tc: 0.40, 0.14–0.65; Vrd_90_: 0.87, 0.74–1.00) showed that Dm and Vrd_90_ variables had a good discriminative ability. The addition of Dm and Vrd_90_ (VL+VM) manifested a good discriminative ability (0.89, 0.75−1.00; 0.89, 0.77−1.00, respectively).

## Discussion

The purpose of this study was to examine the sensitivity of tensiomyographic markers of muscle fatigue in strength-trained men. Our hypotheses were partially fulfilled since Dm could be used as an indirect variable to detect peripheral fatigue and this variable along with Vrd_90_ were the most sensitive TMG indicators. As a result, there was a strong decrease in Dm and Vrd_90_ in both muscles (VL and VM) after a resistance training session (i.e., FG) that resulted in athletic performance impairment (i.e., CMJ height and MIF). However, under a low-fatigue condition (i.e., NFG), no performance and TMG-derived variable showed significant changes. The logistic regression showed a significant model for detecting fatigue, whether it used CMJ height or MIF as reference methods, with only the relative change (from pre- to post) in Vrd_90_ for the VL as a fatigue predictor, presenting good sensitivity and specificity. The determination of the AUC for VL and VM muscles with the CMJ and MIF as the gold standards fatigue test showed that Dm and Vrd_90_ had good to excellent discriminative ability. Therefore, TMG variables, especially Dm and Vrd_90_, could be implemented as tools to measure peripheral muscle fatigue.

Concerning the changes induced by the resistance training session in TMG-derived variables, the fatigued group experienced a decrease in Dm (range: −22.5% to −37.1%) and Vrd_90_ (range: −23.9% to −39.1%). These findings align with Martin-San Agustin et al. (2020), who observed a decrease of 18.2% in the RF, VL, and VM muscles in recreational male athletes. They are also consistent with the results of Kalc et al. (2023), where Dm decreased after both 25% of MVIC (10.2%) and the Wingate test (38.5%) in young healthy males in the VL muscle. Likewise, Wiewelhove et al. (2017) reported decreases in Dm (−8.7%) and Vrd_90_ (−8.6%) in elite male junior tennis players in the RF. It is worth noting that the protocols carried out in those studies were different: 1) Martin-San Agustin et al. (2020) conducted a 60-s fatiguing isometric contraction at 70% MIF in the knee extension exercise; 2) Wiewelhove et al. (2017) examined HIT sessions; 3) Kalc et al. (2023) proposed two different fatiguing exercise interventions: 3-s fatiguing isometric contraction exerting torque equal to 25 % of MVIC and an all-out 30-s cycling test (Wingate), and 4) the present study consisted of 3 x 8 SQ repetitions with 70% 1RM. Acute impairment in Dm after strength training has been associated with decreased muscle function (Hunter et al., 2012), muscle swelling, and exercise-induced muscle damage (Harmsen et al., 2019). Acute decreases in Vrd_90_, which are explained by lower Dm and a longer Tc, also suggest that this variable is sensitive to fatigue produced by exercise, as previously suggested (Mesquita et al., 2023). In fact, Gasparini et al. (2012) defined a decrease of more than 20% in Vrd_90_ during repetitive muscle contractions in individuals with peripheral arterial disease.

The determination of the AUC ± 95% CI for vastus lateralis and vastus medialis muscles with the countermovement jump and maximal isometric force standards fatigue showed that Dm and Vrd_90_ had a good to excellent discriminative ability. Both variables were sensitive to detect peripheral fatigue in VL and VM muscles, at least under fatiguing SQ protocols, compared to reference methods such as the CMJ and MIF. Likewise, the logistic regression showed a significant model (*p* = 0.001) for detecting fatigue, whether it used a CMJ (R^2^_CS_ = 0.582; R^2^_N_ = 0.798) or MIF (R^2^_CS_ = 0.461; R^2^_N_ = 0.624), with only the ∆VL Vrd_90_ variable as a fatigue pattern, with a correct case classification of 84–92%, showing good sensitivity and specificity. These data are different from Wiewelhove et al. (2017), who reported that the sensitivity of the AUC was 33% and the specificity was 50% in Dm and Vrd_90_. These conflicting results may be explained by 1) the protocol, that is, resistance training versus HIT; and 2) the sample under study, elite male junior tennis players versus strength-trained subjects. Nevertheless, our findings align with those of Martin-San Agustin et al. (2020), who proposed that alterations in TMG variables exceeding 0.7 could effectively differentiate between quadriceps experiencing fatigue and those that were not. Additionally, our results are consistent with the observations made by Hunter et al. (2012), who identified significant associations between declines in Dm and impairment in Maximum Isometric Force (MIF) during elbow flexion. This suggests the potential utility of Dm in detecting compromised muscle function. Importantly, Kalc et al. (2023) asserted that the VL TMG variables demonstrated notable diagnostic efficacy in identifying peripheral fatigue during electrically triggered contractions (i.e., AUC Dm 0.74). In contrast, these variables exhibited limited capacity to detect neuromuscular fatigue assessed through voluntary contractions (i.e., AUC Dm 0.42). Notably, De Paula et al. (2015) also observed significant associations between changes in muscle velocity of deformation and MIF in the half-squat following different strength training protocols. The high predictive capacity of athletic performance (i.e., changes in CMJ height and MIF) shown by the changes in Vrd_90_ reinforces the assumption that the decreases in muscle velocity of deformation are related to impaired muscle function. Our results support that Dm and Vrd_90_ could be implemented as tools to measure peripheral muscle fatigue in healthy athletes.

Some limitations of this study design warrant consideration. Primarily, the small sample size may potentially compromise the robustness of the ROC analysis and the acquired data. Nonetheless, we supplied ample information regarding the obtained data and effect sizes, thereby allowing for the discernment of trends in the dataset. Secondly, we only measured two muscles (VL and VM). However, based on Wong et al. (2016), the vastus muscles play an important role during the push-off phase of vertical jumping to maximize jump height and for this reason, we decided to assess both muscles. Thirdly, we cannot extrapolate these results to other groups of people such as females or elite athletes as we could obtain different results and increase or decrease their sensitivity to control muscle fatigue. Fourthly, Abazović et al. (2022) verified the decomposition of the TMG response, effectively isolating the responses of three fiber types (type I, type IIa, type IIb). This approach enables the non-invasive determination of the contribution of each muscle fiber phenotype to the peripheral fatigue of the entire muscle.

## Conclusions

In summary, our results illustrate that Dm and Vrd90, evaluated through TMG, demonstrate heightened sensitivity in detecting fatigue within the VL and VM muscles during resistance training, surpassing conventional methods such as the CMJ and MVIC. As a result, these TMG variables emerge as valuable tools for quantifying muscle fatigue in healthy athletes. Consequently, our study substantiates the initial hypothesis, affirming the heightened sensitivity of TMG in fatigue detection compared to established reference methods. Moreover, the second hypothesis receives partial validation, with both Dm and Vrd90 identified as the most sensitive variables for detecting peripheral fatigue.
